# Rim enhancement on imaging of pancreatic ductal adenocarcinoma: systematic review and meta-analysis of biological and prognostic values

**DOI:** 10.1007/s11547-025-02131-7

**Published:** 2025-10-29

**Authors:** Matteo Renzulli, Alessandro Cucchetti, Valentina Zucchini, Valentina Ciaravino, Cecilia Binda, Cristina Mosconi, Giorgio Ercolani, Emanuela Giampalma

**Affiliations:** 1 Department of Medical and Surgical Sciences – DIMEC, Alma Mater Studiorum – University of Bologna, Bologna, Italy; 2 Morgagni-Pierantoni Hospital, Forlì, Italy; 3 IRCCS Azienda Ospedaliero-Universitaria di Bologna, Bologna, Italy

**Keywords:** Rim enhancement, Pancreatic cancer, Pancreatic neoplasms, Computed tomography, Magnetic resonance, Meta-analysis

## Abstract

Pancreatic ductal adenocarcinoma (PDAC) remains one of the most aggressive malignancies, with limited therapeutic options and poor prognosis. Dynamic contrast-enhanced imaging provides valuable non-invasive information on tumor biology, and rim enhancement (RE) on computed tomography (CT) or magnetic resonance imaging (MRI) has emerged as a potential biomarker of aggressive disease. To clarify its clinical significance, a systematic review and meta-analysis of studies published up to May 31st, 2025, in Medline, Scopus, Web of Science, and the Cochrane Library was conducted. Twelve studies (10 retrospective, 2 prospective) including 2207 patients were analyzed. The pooled prevalence of RE was 36.3%, with a good inter-observer agreement (*κ* = 0.808). RE was consistently associated with reduced resectability (odds ratio [OR] 3.35, 95% confidence interval [CI] 2.19–5.12, *p* < 0.001), poorer histological differentiation (OR 4.23, 95% CI 1.05–17.07, *p* = 0.043), and increased frequency of SMAD4 (OR 1.78, 95% CI 1.22–2.60, *p* = 0.003) and KRAS mutations (OR 2.55, 95% CI 1.37–4.75, *p* = 0.003). Patients with RE also experienced shorter progression-free, disease-free, and overall survival after both surgical and non-surgical treatments (all *p* < 0.001). These findings indicate that RE is a reproducible imaging marker of aggressive tumor biology in PDAC, reflecting unfavorable pathological and molecular features and serving as a predictor of resectability and survival.

**Supplementary Information:**

The online version contains supplementary material available at 10.1007/s11547-025-02131-7.

## Introduction

Pancreatic ductal adenocarcinoma (PDAC) remains one of the deadliest solid malignancies, with a dismal survival despite recent multimodal therapeutic advances [1]. Its lethality stems from multiple converging factors, including a clinically silent onset that delays diagnosis, early systemic dissemination, and resistance to conventional therapies. Accurate prognostic stratification is pivotal for optimizing treatment strategies, yet conventional markers—such as tumor stage, grade, or serum CA19-9 levels—have demonstrated limited sensitivity and specificity in predicting tumor behavior and treatment response [2]. Therefore, additional biomarkers are urgently needed to improve risk assessment and treatment personalization. Increasing attention has been directed toward imaging-derived biomarkers as non-invasive indicators of tumor biology and prognosis.

Pancreatic ductal adenocarcinoma typically appears on CT as a hypodense mass with poor arterial phase enhancement, becoming slightly more conspicuous during the portal venous phase. On MRI, PDAC is usually hypointense on T1-weighted images, variably hyperintense on T2-weighted images, and exhibits restricted diffusion on diffusion-weighted imaging (DWI). Post-contrast dynamic sequences reveal hypoenhancement relative to the surrounding pancreatic parenchyma [3]. A frequently observed imaging feature is rim enhancement (RE), defined by peripheral hyperenhancement with central hypoenhancement, identifiable on both CT [4–10] and MRI [11–15]. Among various radiological features, RE emerged as a potential surrogate of aggressive tumor biology and worse outcomes [8, 13]. Preliminary studies suggested that RE may reflect underlying tumor microenvironments and driver mutations as well as with histological grade, treatment response, and survival [8, 11–13]. Given its accessibility and reliability, RE may serve as a practical biomarker for risk stratification, bridging the gap between imaging and tumor biology.

The present study aimed to highlight the relationship between RE and PDAC resectability, histological characteristics, molecular features and survival outcomes, based on the evidence retrieved through a systematic literature search.

## Methods

### Literature search strategy

We performed a systematic literature search and reported it following the Preferred Reporting Items for Systematic Reviews and Meta-Analyses guidelines. A systematic search of Medline, Scopus and Web of Science (WoS) Core Collection was conducted, searching for articles on rim enhancement in PDAC published until May 31st, 2025. The complete syntax of the search strategy is reported in the Appendix section (in Supplementary Materials). No starting date restriction was applied. No search for grey literature or preprints were attempted. The Cochrane library was also queried. To ensure proper interpretation of the results, the publication language was restricted to English. The review protocol was registered in PROSPERO (CRD420251054071).

### Literature screening and study selection

**Fig. 1:**
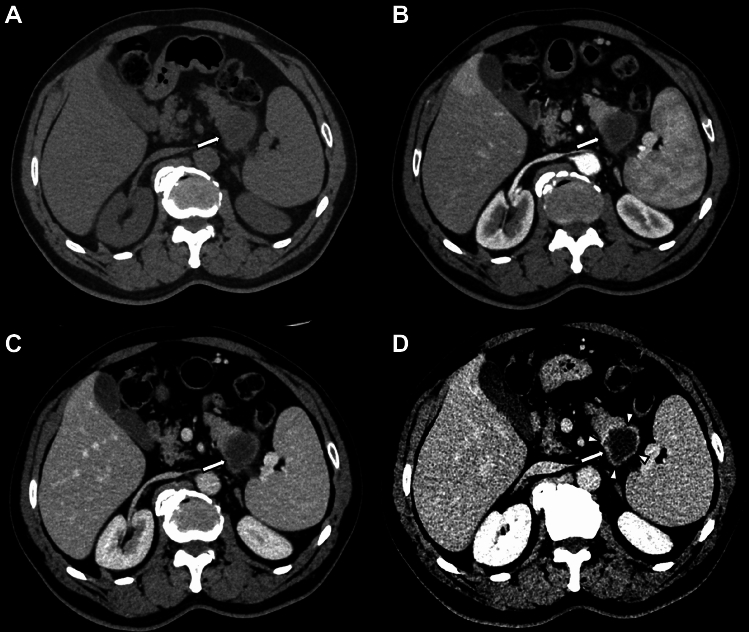
Axial contrast-enhanced CT images of a pancreatic ductal adenocarcinoma at the pancreatic tail (arrows), acquired in unenhanced (**A**), pancreatic (**B**), portal venous (**C**), and delayed (equilibrium) (**D**) phases. In the delayed phase (D), a well-defined enhanced rim (arrowheads) becomes visible, delineating the lesion margin more clearly than in the earlier phases

**Fig. 2:**
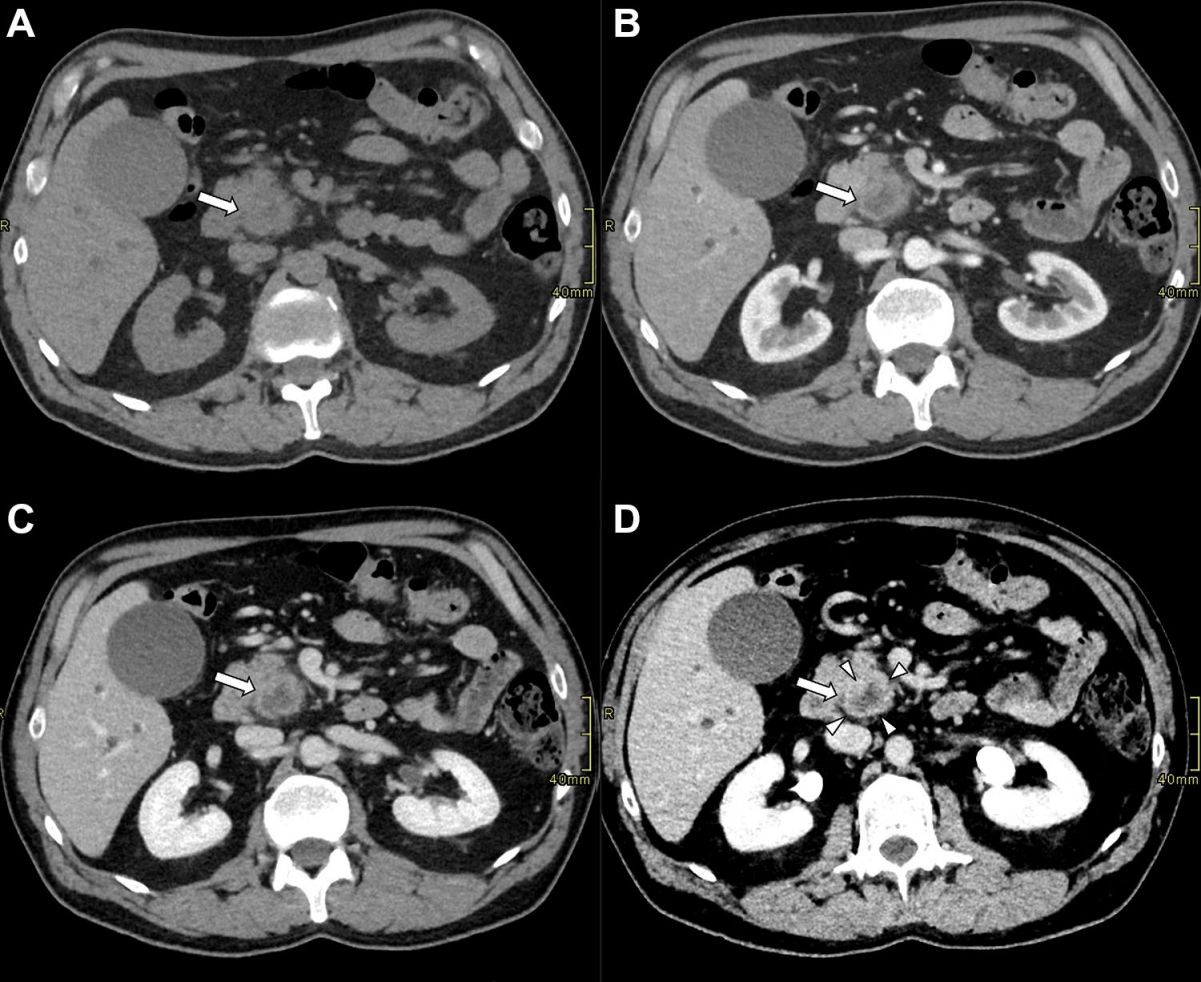
Axial contrast-enhanced CT images of a pancreatic ductal adenocarcinoma at the pancreatic head (arrows), acquired in unenhanced (**A**), pancreatic (**B**), portal venous (**C**), and delayed (equilibrium) (**D**) phases. In the delayed phase (D), a well-defined enhanced rim (arrowheads) becomes visible, delineating the lesion margin more clearly than in the earlier phases

**Fig. 3:**
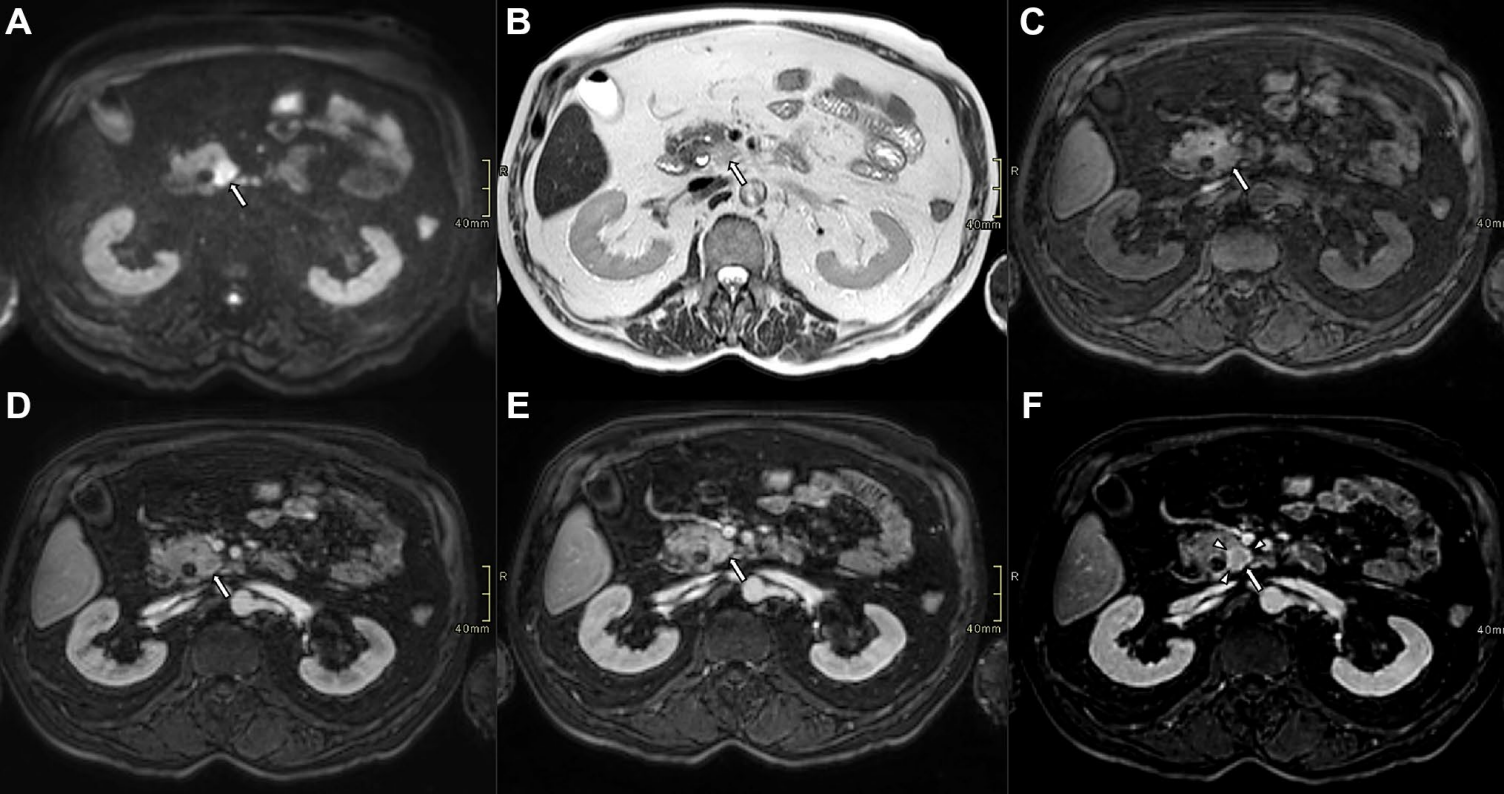
Axial contrast-enhanced MR images of a small pancreatic ductal adenocarcinoma at the pancreatic head (arrows). The tumor demonstrates strong signal restriction on Diffusion weighted image (**A**), hyperintensity on T2-weighted (**B**), and hyperintensity on T1-weighted (**C**) images. The lesion shows disomogeneous enhancement on pancreatic (**D**), and portal venous (**E**) phases. In the delayed phase (D), a well-defined enhanced rim (arrowheads) becomes visible, delineating the lesion margin more clearly than in the earlier phases

**Fig. 4:**
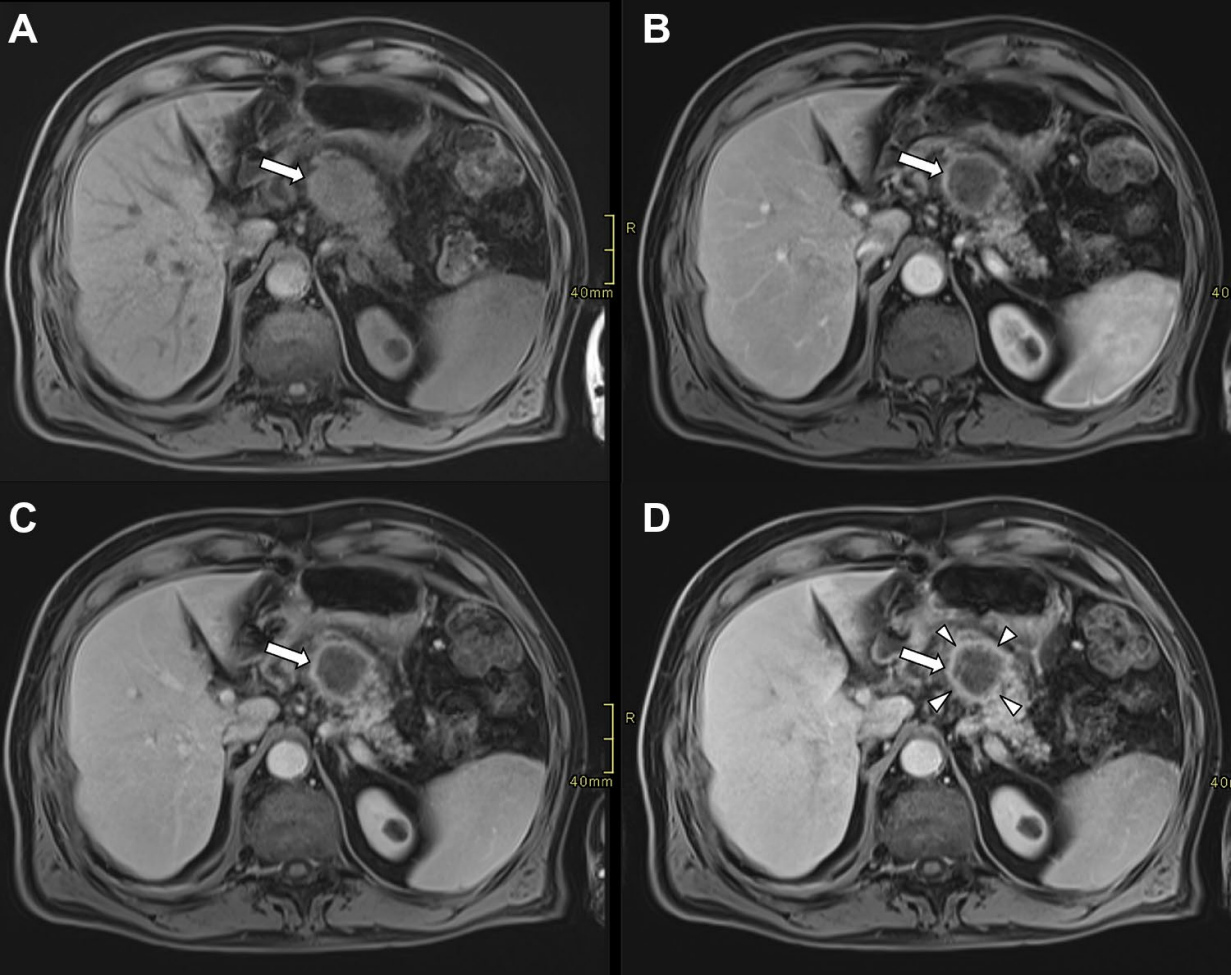
Axial contrast-enhanced MR images of a large pancreatic ductal adenocarcinoma at the pancreatic body (arrows). The tumor demonstrates hypointensity on T1-weighted (**A**) images. The lesion appears hypointense on pancreatic (**B**), portal venous (**C**) and delayed phases (**D**). In the delayed phase (D), a well-defined enhanced rim (arrowheads) becomes visible, delineating the lesion margin more clearly than in the earlier phases

Prospective and retrospective studies exploring imaging features reporting data on rim enhancement at either CT or MRI of histological proven PDAC were included. Rim enhancement was defined as the presence of irregular ringlike enhancement with a relatively hypo-vascular central area on either CT or MRI images (Figs. 1, 2, 3, and 4). After the literature search, the extracted abstracts were screened independently by two reviewers (VC and CM). Full-text screening was performed independently by two reviewers (VZ and CB). In cases in which a study was followed by a more complete study or studies that included the original data set, the most recent, largest or the most complete report was used for the analyses. Such linked studies were identified on the grounds of authorship, institutions and study populations. The reference list of the selected studies was also searched to identify all potentially eligible studies. Any disagreement was resolved by consensus.

### Data extraction

Data extraction was performed on a dedicated spreadsheet by two independent reviewers (VZ and CB). Data included the characteristics of the studies (authorship, publication date, setting, time- period, study design), the characteristics of the included population (type of therapy adopted, type of imaging technique applied, tumor histological size, grading, molecular mutations, progression-free or recurrence-free or disease-free and overall survivals) and the inter-observer agreement on RE assessment. Notably, the included studies adopted very similar definitions of RE, which enhances the comparability of their findings. Two investigators (VC and CM) assessed the quality of each selected study. Considering that no randomized trial could be included due to the nature of the systematic review, the risk of bias of each study was evaluated with the Newcastle–Ottawa Score (NOS) tool. Any disagreement on the risk of bias was resolved by consensus.

### Statistical analysis

Available features were combined using random-effect models. Dichotomous variables were estimated as pooled binomial proportions with a 95% confidence interval (C.I.) applying the Freeman-Tukey double arcsine transformation [16]. When comparing groups, effect size was reported as either odds ratio (OR) or hazard ratio (HR), as appropriate. If necessary, relative risks were converted to HR as per the methods outlined by Parmar et al. [17]. When meta-analyzing inter-observer agreement, the Fisher z-transformation was applied [18]. Studies were not weighted for their quality. Statistical heterogeneity was explored by inconsistency (I^2^) statistics [19]. Analyses were conducted with Stata software (StataCorp. 2024. College Station, TX: StataCorp LP).

## Results

After literature screening and selection (Supplementary Fig. 1 in Supplementary Materials), a total of 12 articles were included in the present study, comprising 2207 patients in whom RE was assessed prior to various therapies [4–15]. The studies’ characteristics are reported in Table 1. Only two studies were prospective [4, 11], and the inclusion periods ranged from 2005 to 2023. Four studies used MRI [11–14], seven used CT [4–10], and one used both imaging techniques [15]. Seven studies included only surgical patients [6, 7, 10, 12–15], three included both surgical and non-surgical patients, one included exclusively chemotherapy-treated patients [5, 8, 11], and one included only patients undergoing radiotherapy [4]. Detailed results derived from subsequent meta-analyses are reported in Table 2.

**Table 1:** Characteristics of the studies investigating rim enhancement (RE) in PDAC included in the systematic review and meta-analysis

Author [Ref]	Year of publication	Study type	Period	N° of patients	N° with RE	N° without RE	% RE	Imaging	Therapy	NOS score
Shen et al. [14]	2025	Retrospective	2016–23	308	162	146	52.6	MRI	Res	7
Choi et al. [11]	2024	Prospective	2019–21	55	12	29	21.8	MRI	Res or CHT	8
Li et al. [12]	2024	Retrospective	2016–22	428	224	204	52.3	MRI	Res	7
Cai et al. [4]	2024	Prospective	2012–19	103	37	66	35.9	CT	RT	9
Yamaguchi et al. [8]	2024	Retrospective	2014–20	158	43	115	27.2	CT	Res or CHT	7
Li et al. [6]	2024	Retrospective	2014–19	97	24	73	24.7	CT	Res	9
Park et al. [9]	2024	Retrospective	2008–19	189	72	117	38.1	CT	CHT	6
Yu et al. [10]	2024	Retrospective	2018–22	150	57	93	38.0	CT	Res	6
Bai et al. [5]	2023	Retrospective	2018–21	502	146	356	29.1	CT	Res or CHT	8
Kim et al. [15]	2022	Retrospective	2012–20	102	41	61	40.2	MRI & CT	Res	8
Lee et al. [13]	2018	Retrospective	2009–14	84	23	61	27.4	MRI	Res	7
Takaji et al. [7]	2018	Retrospective	2005–16	36	18	18	50.0	CT	Res	6

MRI, magnetic resonance imaging; CT, computed tomography; NOS, Newcastle–Ottawa score, Res, resection; PDAC, Pancreatic ductal adenocarcinoma; RE, Rim enhancement

The NOS can be interpreted as follows ≥ 7 = High quality, 5–6 stars = Moderate quality and ≤ 4 stars = Low quality

**Table 2:** Meta-analytic results of inter-observer agreement, tumoral features and survival outcomes among patients with RE-PDAC e non-RE-PDAC

Outcomes	RE-PDAC			Non-RE-PDAC			Effect size (95% CI)	*p*-value
	N. of included patients	Pooled values (prevalences/k-values)	I^2^	N. of included patients	Pooled values (prevalences)	I^2^		
Prevalence	859	36.3% (29.7–43.1)	89.7%	1348	62.4% (55.8–68.8)	89.2%	–	–
*Inter-observer agreement*								
All patients	859	0.808 (0.765–0.850)	62.3%	1348	–	–	–	–
CT	397	0.781 (0.746–0.816)	0.0%	847	–	–	–	–
MRI	462	0.828 (0.744–0.912)	80.5%	501	–	–	–	–
Unresectable disease	189	30.7% (24.2–37.5)	NA	471	12.2% (9.4–15.4)	NA	OR 3.35 (2.19–5.12)	< 0.001
Tumor differentiation	84			203				
Well	–	0% (0.0–3.4)	0.0%	–	24.0% (14.4–35.0)	0.5%	OR 14.77 (2.78–78.45)	0.002
Moderate	–	59.1% (34.4–81.9)	73.0%	–	71.1% (59.2–81.7)	0.0%	OR 2.17 (1.13–4.17)	0.020
Poor	–	38.3% (12.6–67.8)	73.0%	–	12.2% (7.3–18.0)	0.0%	OR 4.23 (1.05–17.07)	0.043
*Molecular features*								
SMAD4 mutation	236	56.8% (50.2–63.3)	NA	233	39.7% (33.4–46.1)	NA	OR 1.78 (1.22–2.60)	0.003
KRAS mutation	174	91.8% (86.8–95.9)	NA	175	79.2% (72.7–85.0)	NA	OR 2.55 (1.37–4.75)	0.003
*Survival outcomes*								
Tumor progression (all)	225	–	–	469	–	–	HR 2.21 (1.69–2.89)	< 0.001
Recurrence (surgery only)	145	–	–	288	–	–	HR 2.17 (1.48–3.18)	< 0.001
Overall Survival (all)	179	–	–	354	–	–	HR 2.21 (1.68–2.91)	< 0.001
Overall survival (surgery only)	64	–	–	122	–	–	HR 2.38 (1.61–3.52)	< 0.001

NA, not computable because of too few studies

OR, odds ratio; HR, hazard ratio; CT, computed tomography; MRI, magnetic resonance imaging; PDAC, pancreatic ductal adenocarcinoma; RE, rim enhancement

I^2^ values indicate heterogeneity levels: 0–40% (low), 30–60% (moderate), 50–90% (substantial), and 75–100% (considerable)

### Prevalence of rim enhancement PDAC

The prevalence of RE-PDAC ranged between 21.8% and 52.6% with a pooled prevalence of 36.3% and high heterogeneity across included studies (I^2^: 89.9%). All studies reported inter-observer agreement, with a pooled *k*-value of 0.808 (95% CI 0.765, 0.850), indicating good agreement (Table 2). The inter-observer agreement was similar using MRI (*k*-value: 0.828) or CT (*k*-value: 0.781; *p* = 0.310).

### Probability of receiving surgery

As reported in Table 2, two studies examined the relationship between RE and the likelihood of undergoing pancreatic surgery, involving 189 patients with RE-PDAC and 471 with non-RE-PDAC [5, 8]. Pooled analysis revealed that pancreatic resection was either not indicated or aborted due to occult metastases in 30.7% of RE-PDACs, compared to 12.2% in non-RE-PDAC cases. This indicates that RE-PDAC was associated with an odds ratio (OR) of 3.35 (95% CI 2.19–5.12; *p* < 0.001) for unresectable disease.

### Histological features

Three studies analyzed histological features in relationship with RE including a total of 84 patients with RE-PDAC and 203 with non-RE-PDAC [7, 8, 13] (Table 2).

The pooled proportion of well differentiated tumors in patients with RE was 0% and was 24.0% in non-RE-PDAC, with an OR of 14.77 (95% CI 2.78–78.45; *p* = 0.002) in favor of non-RE-PDAC. Pooled moderate differentiation was 59.1% in RE-PDACs and 71.1% in non-RE-PDACs with an OR of 2.17 (95% CI 1.13–4.17; *p* = 0.020) in favor of non-RE-PDAC. Finally, the pooled proportion of poorly differentiated tumors was 38.3% in RE-PDACs and 12.2% in non-RE-PDACs, thus, the OR was 4.23 (95% CI 1.05–17.07; *p* = 0.043).

### Molecular features

Three studies analyzed molecular mutations in relation to RE, including a total of 398 patients with RE-PDAC and 379 with non-RE-PDAC [11, 12, 14] (Table 2). Only one study analyzed both SMAD4 and KRAS mutations [11].

The pooled proportion of SMAD4 loss was 56.8% in RE-PDAC and 39.7% in non-RE-PDAC with an OR of 1.78 (95% CI 1.22–2.60; *p* = 0.003). Regarding KRAS mutation the pooled proportion in RE-PDAC was 91.8% and in non-RE-PDAC was 79.2% resulting in an OR of 2.55 (95% CI 1.37–4.75; *p* = 0.003).

### Tumor progression or recurrence

**Fig. 5:**
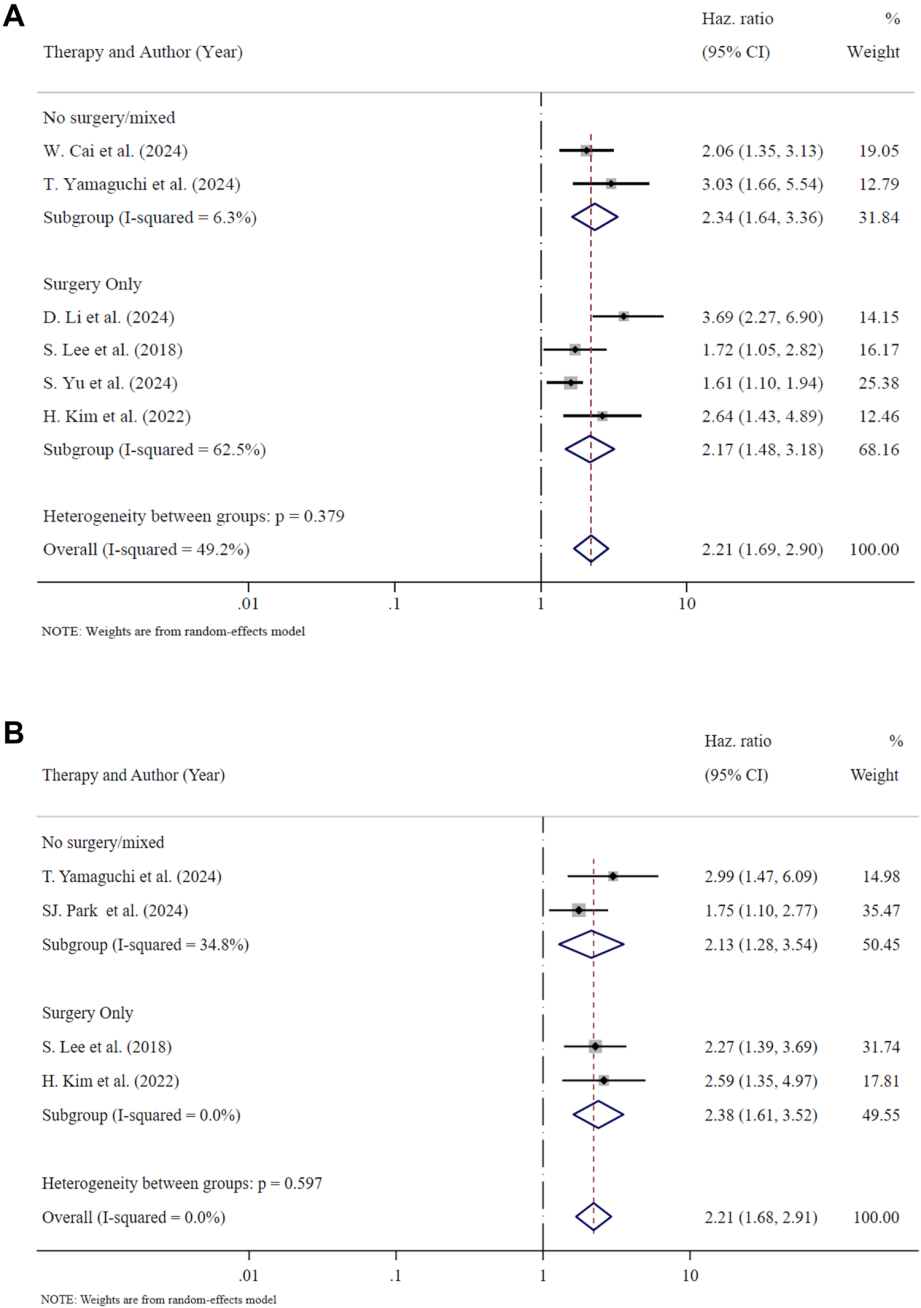
Forest plot on tumor recurrence or progression (Panel **A**) and on overall survival (Panel **B**) stratified by type of therapy received

Six studies assessed the influence of RE on tumor progression or recurrence, analyzing surgical and non-surgical patients, including a total of 225 with RE-PDAC and 469 with non-RE-PDAC [4, 6, 8, 10, 13, 15] (Fig. 5 and Table 2). In all these studies RE resulted as an independent risk factor for the outcome after multivariable regressions, and the pooled HR was 2.21 (95% CI 1.69–2.89; *p* < 0.001).

Four studies reported data on tumor recurrence after surgery, including 145 RE-PDACs and 288 non-RE-PDACs [6, 10, 13, 15], with a pooled HR of 2.17 (95% CI 1.48–3.18; *p* < 0.001), indicating that patients with RE-PDACs have a higher risk of recurrence.

### Overall survival

Four studies analyzed the relationship between RE and overall survival in surgical and non-surgical patients, including a total of 179 patients with RE-PDAC and 354 non-RE-PDACs [8, 9, 13, 15] (Fig. 5 and Table 2). In all these studies RE resulted as an independent risk factor for death after multivariable regressions, and the pooled HR was 2.21 (95% CI 1.68–2.91; *p* < 0.001).

Two studies reported data on overall survival after surgery, including 64 RE-PDACs and 122 non-RE-PDACs [13, 15] with a pooled HR of 2.38 (95% CI 1.61–3.52; *p* < 0.001), indicating worse post-operative survival in patients with RE-PDACs.

## Discussion

This systematic review and meta-analysis showed that RE on contrast-enhanced imaging is not merely a descriptive radiologic pattern but a reproducible and clinically meaningful imaging biomarker of aggressive PDAC biology. With a pooled prevalence of 36.3% and good inter-observer agreement (*k* = 0.808), RE was shown to be both frequent and reliable across imaging modalities utilized for diagnosis and staging of PDAC, such as CT and MRI, making it a practical candidate for inclusion in routine clinical assessment of PDAC characteristics [5, 8, 11, 13, 14, 20–22].

Since surgery remains the only potentially curative treatment for PDAC [23], the first consideration must be the likelihood of achieving resectability. Bai et al. studied the CT features that could predict occult metastases (OM) at surgical exploration of 502 patients with PDAC [5]. They observed that RE was more frequent in the OM group (52.9%) than in the non-OM group (25.3%; *p* < 0.001). This correlation was confirmed at multivariable analysis, where RE-PDAC provided an OR of 4.66 (95% CI 2.61–8.32; *p* < 0.001) for OM at surgical exploration. Subsequently, Yamaguchi et al. used CT in 158 patients with PDAC reporting that RE was more frequent in 52 patients who did not finally receive surgery (44.2%) than in 106 patients who received surgery (18.9%; *p* < 0.001) [8]. These studies finally suggested that detecting RE on CT justifies additional staging with contrast-enhanced liver MRI or whole-body FDG-PET/CT. These advanced imaging modalities can improve detection of distant metastases, thereby preventing unnecessary upfront surgical exploration.

The non-resectability of PDAC is the obvious epiphenomenon of a more aggressive tumor which leads to early local invasion or extra-pancreatic spread. Lee et al. studied RE on preoperative MRI in 143 resected patients and correlated it with histological features [13]. None of the 23 RE-PDACs were well differentiated tumors and 52.2% were poorly differentiated. This was markedly different from the 61 non-RE-PDACs, among which 16.4% were well differentiated and 67.2% were moderately differentiated (*p* = 0.002). Tumor necrosis was present in 56.5% of RE-PDACs and in 14.8% of non-RE-PDACs (*p* < 0.001), with significant different amount of remaining acini (*p* = 0.008). Takaji et al. using CT in small PDAC (≤ 20 mm) did not observe well differentiated tumors in 18 patients with RE, whereas in 27 without RE the prevalence was 25.9% (*p* = 0.031) [7]. Also, Yamaguchi et al. using CT in 106 patients submitted to surgery did not observe well differentiated tumors in RE-PDACs, being mostly poorly differentiated (55%). On the contrary, non-RE-PDACs had well differentiated tumors in 26% and poorly differentiated tumors in only 9% (*p* < 0.001) [8]. Notably, Takaji et al. reported that RE prevalence was up to even in small PDACs, indicating that this aggressive imaging feature can manifest ab initio during tumor growth.

From a molecular perspective, KRAS and SMAD4 represent the most extensively characterized driver mutations in PDAC. While KRAS mutations are reported to occur in approximately 85% of cases, SMAD4 loss is usually reported to occur in about 50% of tumors [24, 25]. In the study from Li et al., RE on preoperative MRI emerged as significantly associated with SMAD4-mutated PDAC [12]. Specifically, SMAD4 loss was observed in 59.4% of 224 RE-PDACs compared to 44.6% in 204 non-RE-PDACs (*p* = 0.002). The study from Choi et al. conducted on 12 patients with RE-PDACs and 29 non-RE-PDACs on MRI, showed that KRAS variant allele frequency (VAF) was higher in RE-PDACs (median 27.3%; IQR: 20.8, 35.7) than in non-RE-PDACs (median 13.2; IQR: 6.3, 25.4; *p* = 0.008) [9]. Conversely, KRAS-mutation was similar in RE-PDACs (100%) and non-RE-PDACs (86.2%; *p* = 0235) as well as SMAD4 mutation that was 8.3% in RE-PDACs and 10.3% in non-RE-PDACs (*p* = 0.813). Finally, Shen et al. (2025) investigating MRI features associated with KRAS mutation in 308 surgically confirmed PDAC, observed that RE was present in 56.2% of KRAS-mutated PDACs and in 34.0% of non-KRAS-mutated tumors (*p* = 0.040) [14]. Thus, RE-PDACs present more frequently molecular alterations (KRAS up to 92% and SMAD4 up to 57%), in line with the progression and the development of invasive PDAC [25]. How KRAS mutations can be effectively targeted by KRAS-inhibitors or whether SMAD4 can be targeted remains an open issue [24, 25].

All these characteristics finally converge to worse prognosis, both without as well with surgery. Crucially, across all studies analyzed RE consistently emerged as an independent prognostic factor in multivariable analyses for progression-free, recurrence-free, and ultimately overall patient survival [4, 6, 8–10, 13, 15]. Rim enhancement correlated with worse prognosis also after FOLFIRINOX chemotherapy which represents to date the most effective chemotherapy. Park et al. reported that RE was correlated with an HR for overall survival at multivariable analysis of 1.75 (95% CI 1.10–2.77; *p* = 0.018) [9]. This aspect should be considered together with consideration made for the likelihood of not receiving surgery because of occult metastases in the setting of anatomically resectable patients. The National Comprehensive Cancer Network (NCCN) guidelines suggest that in presence of localized anatomically resectable PDAC, neoadjuvant should be considered in patients at high-risk of developing an early cancer recurrence (e.g., elevated CA19-9, large tumors, nodal involvement, or severe symptoms) [26]. Based on current evidence, it can be suggested that RE-PDAC may be classified as biologically borderline, and neoadjuvant therapy in these patients could serve as a test to detect emerging metastases or progressive local disease, thereby avoiding futile upfront surgery [27].

The present study has several limitations related to the quality of the included studies. Most studies were not specifically planned to directly compare RE-PDACs to non-RE-PDACs and only four studies were specifically designed with this aim [5, 8, 11, 13]. However, it should be noted that most of the studies included had high quality when assessing the Newcastle–Ottawa Score (9 out of 12). Furthermore, for non-resectability and molecular mutations, only two studies were available for the analysis. Data were also insufficient to examine potential relationships between RE and tumor location, size, or CA19-9 levels. Finally, we acknowledge that for certain parameters, such as the prevalence of rim-enhancement and inter-observer agreement, heterogeneity was relatively high. This was not unexpected given the nature of the present meta-analysis, which pooled data from studies with varying designs and populations, as highlighted before. While meta-regression or subgroup analyses could ideally be used to further investigate the sources of heterogeneity, the limited number of available studies unfortunately precludes such analyses. These limitations collectively suggest that future studies should adopt a comparative approach between RE-PDAC and non-RE-PDAC, together with a more comprehensive data collection and comparison. Despite these limitations, RE remains a clinically valuable feature due to its high inter-observer agreement and ease of assessment. In an era dominated by complex radiomics and time-consuming deep learning approaches, this straightforward imaging characteristic can bring practical utility in clinical decision-making, as discussed before.

In conclusion, rim enhancement is a readily observable, reliable, and prognostically relevant imaging feature in PDAC. It is strongly associated with adverse histological features, aggressive molecular alterations, and inferior survival outcomes, both with and without surgical intervention. Given its ease of use, RE could be evaluated for possible integration into clinical staging systems and future definitions of biologically borderline PDAC through appropriately designed prospective studies. Such studies may clarify whether its incorporation into treatment algorithms can improve risk stratification and support more individualized oncologic care.

## Supplementary Information

Below is the link to the electronic supplementary material.

Supplementary file1 (DOCX 36 kb)
