# Supplementary material for: Rim enhancement on imaging of pancreatic ductal adenocarcinoma: systematic review and meta-analysis of biological and prognostic values
Source: Radiol Med. 2025 Oct 29;131(2):167–76. doi: 10.1007/s11547-025-02131-7 (PMC12932284; doi:10.1007/s11547-025-02131-7)

**Supplementary Information**

**Medline:** ("rim enhancement"[Title/Abstract] OR "peripheral enhancement"[Title/Abstract]) AND ("imaging"[Title/Abstract] OR "MRI"[Title/Abstract] OR "CT"[Title/Abstract] OR "radiology"[Title/Abstract]) AND ("pancreatic neoplasms"[MeSH Terms] OR "pancreatic cancer"[Title/Abstract] OR "pancreatic adenocarcinoma"[Title/Abstract])

Number of articles retrieved = 34

**Scopus:** TITLE-ABS-KEY ("rim enhancement" OR "peripheral enhancement") AND TITLE-ABS-KEY (imaging OR mri OR ct OR radiology) AND TITLE-ABS-KEY ("pancreatic cancer" OR "pancreatic adenocarcinoma" OR "pancreatic neoplasm*" OR "pancreatic ductal adenocarcinoma")

Number of documents retrieved = 33

**Web of Science:** TS=("rim enhancement" OR "peripheral enhancement") AND TS=(imaging OR MRI OR CT OR radiology) AND TS=("pancreatic cancer" OR "pancreatic adenocarcinoma" OR "pancreatic neoplasm*" OR "pancreatic ductal adenocarcinoma")

Number of documents retrieved = 19

**Supplementary Figure 1:** Literature screening and selection.


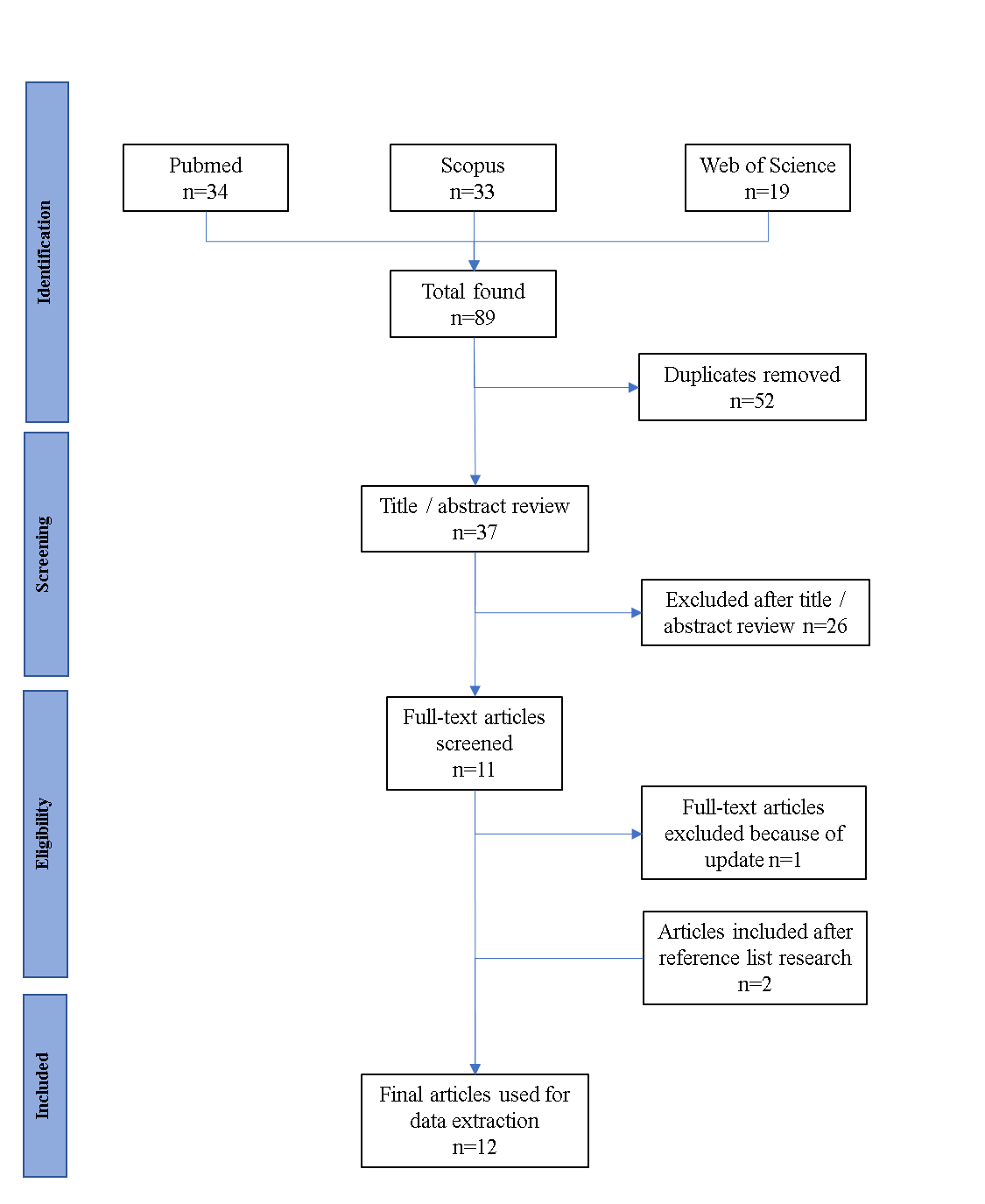

Supplement: Supplementary file 1 — Supplementary file1 (DOCX 36 kb) [file 11547_2025_2131_MOESM1_ESM.docx]
